# Trauma and psychosocial adversity in youth with autism spectrum disorder and intellectual disability

**DOI:** 10.3389/fpsyt.2024.1322056

**Published:** 2024-01-26

**Authors:** Sarah J. Palmer, Yael Dvir

**Affiliations:** Department of Psychiatry, University of Massachusetts Chan Medical School, Worcester, MA, United States

**Keywords:** autism spectrum disorder, intellectual disability, trauma, post-traumatic stress disorder, child and adolescent psychiatry

## Abstract

Traumatic experiences contribute significantly to behavioral and mood dysregulation syndromes presenting for treatment to behavioral health settings. Individuals with Autism Spectrum Disorder (ASD), Intellectual Disability (ID) and developmental delay experience traumatic events more frequently than their typically developing peers. However, measures used to identify trauma related disorders and treatment thereof are based on typically developing individuals. Regardless of the baseline characteristics of individuals who experience trauma, trauma exposure is the result of multiple interdependent environmental, social, and familial characteristics. We used the “ecological systems analysis approach” to structure our review of the impact of trauma on those with ASD and ID. In addition, the COVID-19 pandemic which exposed the global population to a collective trauma, has also catalyzed investigations into the challenges faced by members of society most dependent on social services. Children with ASD and ID were among those vulnerable individuals, and the COVID-19 pandemic has allowed researchers to better understand the impact of a collective trauma on those individuals. It is imperative that we understand current research and recommendations for identifying and treating trauma-related disorders in individuals with developmental disorders to best inform clinical practice and directions for future research in this area.

## Introduction

Developmental disability impacts individuals in multiple spheres of social, educational, vocational, and personal functioning. Presentations of developmental disabilities are diverse, and rates in the United States have consistently increased in recent years (1). Intellectual disabilities (ID) are defined by deficits in intellectual functions and adaptive functioning with onset during childhood (2) While there are multiple clinical presentations of ID, the DSM-V categorizes ID into four severity levels (mild, moderate, severe, and profound) and three domains (conceptual, social, and practical) (2).

Autism spectrum disorder (ASD) is a type of developmental disability that is of particular interest due to its increasing prevalence and significant overlap with ID (3). The prevalence of ID among individuals with ASD is reported to be 22.9 (95%CI [17.7-29.2]) (4). ASD is characterized by persistent deficits in social communication and social interaction across multiple contexts resulting in 1. Deficits in social-emotional reciprocity, 2. Deficits in nonverbal communicative behaviors used for social interaction and 3. Deficits in developing, maintaining, and understanding relationships (2). Criteria for ASD also includes restricted repetitive patterns of behavior, interests, or activities (Ibid).

Trauma-related disorders including Post Traumatic Stress Disorder (PTSD) require exposure to a trauma to meet criteria for diagnosis (2). Per the DSM-V, for individuals older than six years of age, trauma is defined as exposure to actual or threatened death, serious injury, or sexual violence. For individuals younger than six years of age, trauma can include the knowledge that actual or threatened death, serious injury or sexual violence occurred to a caregiver (2). Outside of the definitions laid out by the DSM-V, it has been shown clinically that chronic neglect of basic needs, especially during early developmental periods, can lead to a PTSD presentation (5). Broadly, child maltreatment encompasses multiple categories of abuse and neglect, including physical assault, psychological aggression, and neglect (6), or family interpersonal violence (IPV), nonfamily IPV, non-IPV trauma, separation/loss, acts of commission, acts of omission, contact trauma and noncontact trauma (5). Providers have posited concerns that events that may seem trivial to others can be traumatic for individuals with ASD and ID (7–9). Given the broad definitions of trauma and maltreatment and the impact of multifactorial individual vulnerability on the risk of developing a trauma associated disorder, this review will discuss the impact of traumatic experiences on those with ASD and ID more broadly.

Exposure to trauma and maltreatment is higher among children with intellectual disability (10) (11). A 2019 population-based sample using a linked cohort between the Department of Social Services and the Autism and Developmental Disabilities Monitoring (ADDM) network found that a diagnosis of ASD alone increased the risk of maltreatment with an OR of 1.86 [1.36, 2.52]; a diagnosis of ASD and ID increased the risk further with an OR of 2.35 [1.77,3.12]; and a diagnosis of ID alone increased the risk of maltreatment the most with an OR of 2.45 [2.09, 2.88] (12). Authors considered the possibility that this discrepancy highlights the concerns that deficits in social communication in children with ASD, and/or possible biases among caseworkers may present additional challenges to detecting maltreatment in this vulnerable population (12, 13). Elsewhere, the possibility that ASD-associated deficits in social cognition including naivete, poor social boundaries, and difficulty detecting a violation of social rules or inappropriate behavior may also contribute to higher risks of victimization while also increasing their vulnerability to interpersonal manipulation which may also be a barrier to reporting maltreatment in this population (14). Despite the prevalence of maltreatment in this population, the co-occurrence of PTSD with ASD and ID has not been well described in the literature (4, 8, 14, 15).

In their 2011 paper authors Algood et al. presented a review on the maltreatment of children with developmental disabilities using an ecological systems analysis. Ecological systems analysis allows maltreatment to be understood as an outcome of a complex and interactive set of interdependent systems. Algood’s review is organized by systems the following way: Social-demographic characteristics, Age, Gender, Special Education, Microsystem, Parent-Child Relationship, Domestic violence, Ecosystem, Parenting Stress, Parents’ Social Support, Area of Residence, and Macrosystem. Seeing as children with ASD and ID account for a significant number of those receiving community services and educational supports and constitute a large portion of those most vulnerable to maltreatment, authors concluded that contemporaneous policies impacting systems caring for children with disabilities must also consider their impact on the ecosystem which predisposes this vulnerable population to abuse (16, 17).

The Centers for Medicaid and Medicare Home and Community Based (HCBS) Settings Rule was passed in 2011, stipulating that community-based long-term services and support be provided to individuals meeting criteria for developmental disabilities, and that states must follow this rule by March 17, 2023 (Autismsociety.org, accessed 8/11/2023). Between 2011 and 2023, investigations into the trends of adversity faced by individuals with ASD and ID progressed. In addition, the COVID-19 pandemic which presented a global challenge transecting the ecological domains has given researchers the opportunity to study the interplay of those domains. Therefore, we present an updated ecological analysis of maltreatment faced by children with developmental disabilities, and specifically ASD, to help guide clinical practice and future policy decisions.

## Socio-demographic characteristics

In a 2023 secondary analysis of characteristics of children with ASD using existing medical records in the Autism and Developmental Disabilities Monitoring Network (ADDM Network) collected in 2020, the Centers for Disease Control and Prevention reported that overall, ASD prevalence per 1,000 children aged eight years in the study population was 27.6 or one in 36, with overall ASD prevalence of 43.0 among boys and 11.4 among girls. The prevalence of ID alone was reported to be 11.8 per 1,000 and 37.9% of children with ASD aged eight years and above were classified as having intellectual disability as noted in their developmental evaluation by a qualified professional, or an intellectual quotient (IQ) score ≤ 70 (3).

This data also indicated that prevalence of ASD in children aged eight years in the ADDM Network differed among racial and ethnic groups. Prevalence of ASD among white children (24.3) was lower than prevalence among Black, Hispanic, or Asian/Pacific Islander children (29.3, 31.6, and 33.4, respectively) (3). Additionally, girls with ASD were more likely to be classified as having ID compared with boys with ASD (42.1% versus 36.9%), and Black children were more likely than Hispanic and white children to be classified as having intellectual disability (50.8%, 34.9%, and 31.8%, respectively) (3).

Socio-economic status also has an impact on the ability of families to manage stress. During the COVID-19 pandemic it was found that pre-pandemic poverty was significantly directly linked to caregivers’ emotional distress, and employment decrease was significantly directly related to household children’s behavioral problems (18).

## Special education

Special education is a critical component of treatment and offers important support to families with children with ASD. Therefore, it is not surprising that withdrawal of these supports during the COVID-19 Pandemic resulted in increased difficulties for children and their families. Latzer et al. surveyed 31 families in Israel with children with ASD who lost access to a specialized education system offering full day classes six days per week during COVID-19 lockdown. While the survey used did not specifically ask about maltreatment or abuse, all parents surveyed indicated that they did not have the knowledge or means to provide for their children’s developmental needs without the professional support offered by school. The loss of expertise, therapies, physical space, and changes to routine contributed to increased repetitive behaviors and developmental regression, overall increasing the difficulty experienced by families (19).

The strain on families caring for children with ASD alone when services had previously been provided by a team of professionals could have significant consequences for the child’s experience of maltreatment. In April 2020, investigators in Hong Kong surveyed 417 children with special education needs (SEN) and 25,427 typically developing (TD) children studying at mainstream schools (6). Among the children with SEN, 19.18% had physical disabilities, 20.38% had ID, 45.8% had mental disorders (e.g. ASD, attention deficit hyperactivity disorder), 24.22% had other disabilities including global developmental delay, isolated significant delay in motor/language skills, or syndromal/genetic disorder, 7.91% had visual impairments and 5.76% had hearing impairments (6). During COVID-19 school closures, investigators found that children with special education needs had significantly more emotional and behavioral difficulties across all aspects than typically developing peers (p<0.01) and experienced poorer overall quality of life (68.05 vs. 80.65, *p*< 0.01) (6).

While rates of child maltreatment in typically developing children were not reported by authors, they found that 23.5% of children with SEN had at least one episode of severe physical assault and 1.9% experienced very severe physical assault, while 80.5% were victims of psychological aggression and 28.7% suffered from neglect during the pandemic (6). Compared to maltreatment prior to the pandemic, relative risk of physical assault among SEN children was 1.19 ( $\chi^{2}$ = 9.938, *p*=0.01) and psychological aggression was 1.50 ( $\chi^{2}$ = 54.604, *p*=0.01) (6). While it has been reported that risk of child maltreatment (CM) increased for all children during the pandemic, the epidemiological measurement of CM rates has been complicated by a decrease in CM allegations due to school closures during the pandemic and loss of contact with mandated reporters in the education system, and there are no studies on this topic that exclude children with ASD or ID (20). The ability to compare rates of CM among TD children and children with ID and ASD is therefore limited. However, the risk of maltreatment among children with SEN increased significantly during the pandemic (6).

**Microsystem “The relations between the developing person and environment in a direct setting where the person is embedded” – (21).

## Parental stress and parent-child relationship

Studies of parent-child relationships are limited as they focus on heterosexual couples and require parents and children to live together in order to be included, which excludes families where parents live separately or who are not in heterosexual relationships (22, 23). Despite this, understanding the current literature is useful in understanding family dynamics as a significant factor in exposure to domestic violence.

A 2020 study set out to examine the relationship between parenting stress and the emotional quality of the parent-child relationship using the Five-Minute Speech Sample (FMSS) in 150 families of children with ASD aged 5-12 years (23). Parenting stress was measured using the Burden Interview and results indicated that parenting stress and depressive symptoms in mothers were negatively associated with FMSS Warmth and positively associated with FMSS Criticism toward the child with ASD. In fathers, FMSS Warmth toward the child with ASD was negatively associated with mother’s level of parenting stress (Ibid). While fathers’ FMSS Warmth was correlated to mother’s level of parenting stress, mothers’ FMSS Warmth was not mediated by fathers’ parenting stress. In this study, authors also reported that 20-56% of parents of children with ASD report a clinically significant level of depressive symptoms relative to 7-29% of parents with children with other types of disabilities, and 8-19% of parents of typically developing children (23). A 2014 investigation of parent-child relationship quality and parental depression in heterosexual couples which did not control for the presence of ASD or ID, showed that while mothers’ depressive symptoms were associated with lower father-child relationship quality, father’s daily depressive symptoms were associated with higher mother-child relationship quality (22). It is notable that father-child relationship stress was consistently shown to be mediated by mother’s level of stress or depressive symptoms regardless of the presence of ASD in the child, yet father’s stress does not impact the mother-child relationship the same way (22, 23).

While there may be common trends in the dynamics between families of children with and without ASD, it is well established in the literature that parents of children with ASD report higher levels of parenting stress when compared with parents of typically developing children (23). In their 2021 study, Hickey et al. demonstrated that mother and father level of parenting stress is positively correlated to both parents’ ratings of ASD symptoms and behavior problems and father level of parenting stress is significantly higher if the child with ASD is male and if the father is white, non-Hispanic (23).

Of note, while ID co-occurred in 34.4% of the 150 participating families in the 2021 study by Hickey et. al., no additional analysis was provided comparing parental stress in this subgroup. It has been previously shown that families of children with autism report significant parental stress at a rate of at least 45%, more than double the rate reported by families of children with other developmental disabilities. (24).

In the context of the COVID-19 pandemic, parental stress continued to be reported as higher among parents of children with SEN. In the 2020 Hong Kong study, parents of children with SEN reported significantly higher parental stress compared to parents of TD children (46.41 vs. 43.36, *p*< 0.01) (6). This trend was shown again in a 2022 study that compared parental stress before COVID-19 and during among families with young children, utilizing reference samples for comparison using data collected by the Berlin-based market research company INFO Marktforschungsinstitut (25). While parental stress was generally significantly higher than pre-COVID-19 levels (*M*=36.93, SD=10.45, range 18-71 vs. pre-COVID-19 *M*=34.72, SD = 10.63, range = 18 = 70; *t*(1023) = 12.474, *p*<0.001) with a small effect size (*d*=0.21), it was significantly lower than parental stress in a clinical reference sample of parents in treatment for their child’s behavior problems (a population included in children with SEN) where parental stress was measured *n*=51, *M*=43.2, SD=9.1 (6, 25).

**Exosystem “interactions between two or more settings, of which one is the immediate setting”- (21).

During the COVID-19 pandemic, parents took on the role of full-time educators, caregivers, while managing jobs, financial stress, and their own health challenges (19). Studies investigating trends in parental stress and occurrence of adverse childhood experiences (ACE) showed that of the 6.5% of families surveyed who reported a lifetime occurrence of ACE, 34.8% reported an increase in occurrence during the pandemic (17.6% no change, 47.5% decrease) with the highest lifetime occurrence for children witnessing domestic violence (n=332, 32.4%) and for verbal emotional abuse against children (n=332, 32.4%) (25). Parents who reported an increase in ACE also reported higher pandemic-related stress, poorer parental outcomes, with the largest effect sizes observed for parental stress (25). Further investigation of parental stress and trends in domestic violence show that inability to meet financial obligations and loss of social supports during the COVID-19 pandemic are most associated with increase report of family stress and domestic violence (26).

## Domestic violence

As described in the introduction, children with ASD and ID experience significantly higher rates of reported and substantiated maltreatment, including exposure to domestic violence, when compared to their typically developing peers (12).

There is evidence to suggest that the COVID-19 pandemic increased occurrences of domestic violence in families of typically developing children and those raising children with ASD (26). Lockdowns, school closures, and social distancing presented unique challenges to families during the COVID-19 pandemic. Utilizing the Canadian Perspective Survey Series, Beland et al. surveyed 4,627 individuals to better understand the mechanism contributing to domestic violence trends during the COVID-19 pandemic. Authors showed that a family’s level of concern about their ability to meet financial obligations and essential needs and need to maintain social ties were most positively associated with concerns regarding domestic violence and family stress from confinement (26). In a study conducted in Italy, 25% of parents surveyed reported that one parent had to quit their job (26.1% of mothers, 27.5% of fathers) to take care of their child with ASD and 94% of study participants reported that the COVID-19 pandemic was financially difficult for them (27).

During the COVID-19 pandemic, caregivers of children with special healthcare needs exhibited more emotional distress and reported higher levels of household children’s behavioral problems than caregivers of children without special healthcare needs (18). Tso and colleagues found that increased parental stress during the COVID-19 pandemic was associated with increased likelihood of child maltreatment in children with SEN including psychical assault (*r*=0.237, *p*<0.05), severe physical assault (*r*=0.195, *p*<0.05), psychological aggression (*r*=0.363, *p*<0.01), and neglect (*r*=0.293, *p*<0.01). Authors reported that when comparing maltreatment rates in SEN children prior to the COVID-19 pandemic, they found significant increases in the rates of physical assault (59.8% vs. 71.2%, *p*<0.01) and psychological aggression (53.7% vs 80.5%, *p*<0.01) during the COVID-19 pandemic (6).

## Parents’ social support

Parental wellbeing informs the risk of childhood maltreatment and parents of children with ID or ASD rely on community resources, school, medical professionals, and therapies in order to maintain routine, improve functioning in the community, and allow parents greater satisfaction in their relationships with their children. Access to these services can be limited by availability, affordability, transportation, among other factors which were all exacerbated by the COVID-19 pandemic limiting availability of in-person supports to families of children with SEN.

In many cases, families lost their entire support systems during the COVID-19 pandemic (19, 28). A global review consistently showed that loss of services and support systems increased parental stress for parents of children with ASD, and that accessing alternative support systems through relatives, or through emergency health services, allowed parents to reduce their psychological distress (28). In contrast, a 2021 national survey of US families, found that while emotional social support was correlated to reduced caregiver emotional distress and decreased behavioral problems for children, this effect was not observed in households with children who have special healthcare needs (18).

## Identifying post-traumatic stress disorder in children with ASD

Exposure to trauma is not commensurate with a trauma reactive disorder, such as PTSD. In the general population, PTSD has a prevalence of approximately 6-8%, and in groups with high exposure to severe psychological trauma, such as combat veterans, refugees, and victims of assault, its prevalence can reach 25% (29). PTSD is also not the only psychiatric sequela of trauma, as individuals may go on to develop other disorders including reactive attachment disorder, disinhibited social engagement disorder, acute stress disorder as well as dissociative and adjustment disorders following a traumatic experience (2). Clinically observed sequelae of trauma in children has also been understood to present as developmental trauma disorder (DTD), a diagnosis not included in the DSM-V but understood to integrate developmental psychopathology, attachment and relational capacity, emotion, and intellectual functioning of the child in the setting of multiple trauma exposures during the early developmental period (5). For the purposes of this review and treatment recommendations, we will focus on PTSD identification and treatment.

The diagnosis of PTSD requires that an individual meet diagnostic criterion and depends on the evaluator’s ability to elicit a history supporting an appropriate diagnosis. The DSM-5 criteria for PTSD include exposure to actual or threatened death, serious injury, or sexual violence; at least one intrusion symptom associated with the traumatic event, persistent avoidance of stimuli associated with the traumatic event, negative alterations in cognitions and mood associated with the traumatic event, and marked alterations in arousal and reactivity associated with the traumatic event for a minimum of one month (2). In children six years of age or younger, exposure can include learning that a traumatic event occurred to a parent or caregiving figure, and negative alterations in cognitions include constriction of play and reduction in the expression of positive emotions (2).

ASD is characterized by persistent deficits in social communication and social interaction across multiple contexts (2). Given these deficits in social communication, individuals with ASD may be challenged to describe the symptoms and history required to be diagnosed with PTSD (13). The diagnosis of PTSD in children with ASD, therefore, requires careful clinical observation and understanding of how the symptoms described in the DSM-5 criteria will present in these individuals. Rating scales and structured diagnostic interviews may aid in the proper diagnosis of PTSD. For example, the Child PTSD Symptom Scale for DSM-5 (CPSS-5) is a 27 item scale intended to reveal the presence of PTSD symptoms in children ages 8-18 based on self-report by the child or a caregiver and is based on the DSM-5 criteria (30). This scale is limited by the ability of the child or caregiver to accurately complete the scale and depends on their ability to accurately identify and report symptoms, which requires emotional awareness and verbal abilities that are often deficient in children with ASD (13). In practice, when assessing an individual with ASD who has experienced trauma, it is imperative to speak clearly and slowly with a gentle tone, and to frame questions in a way that they are accessible to the patient so as not to increase anxiety. Part of this effort may be to utilize all options for assistance with communication including American Sign Language, visual cues, professional liaison, or tablet (14).

**Figure 1:**
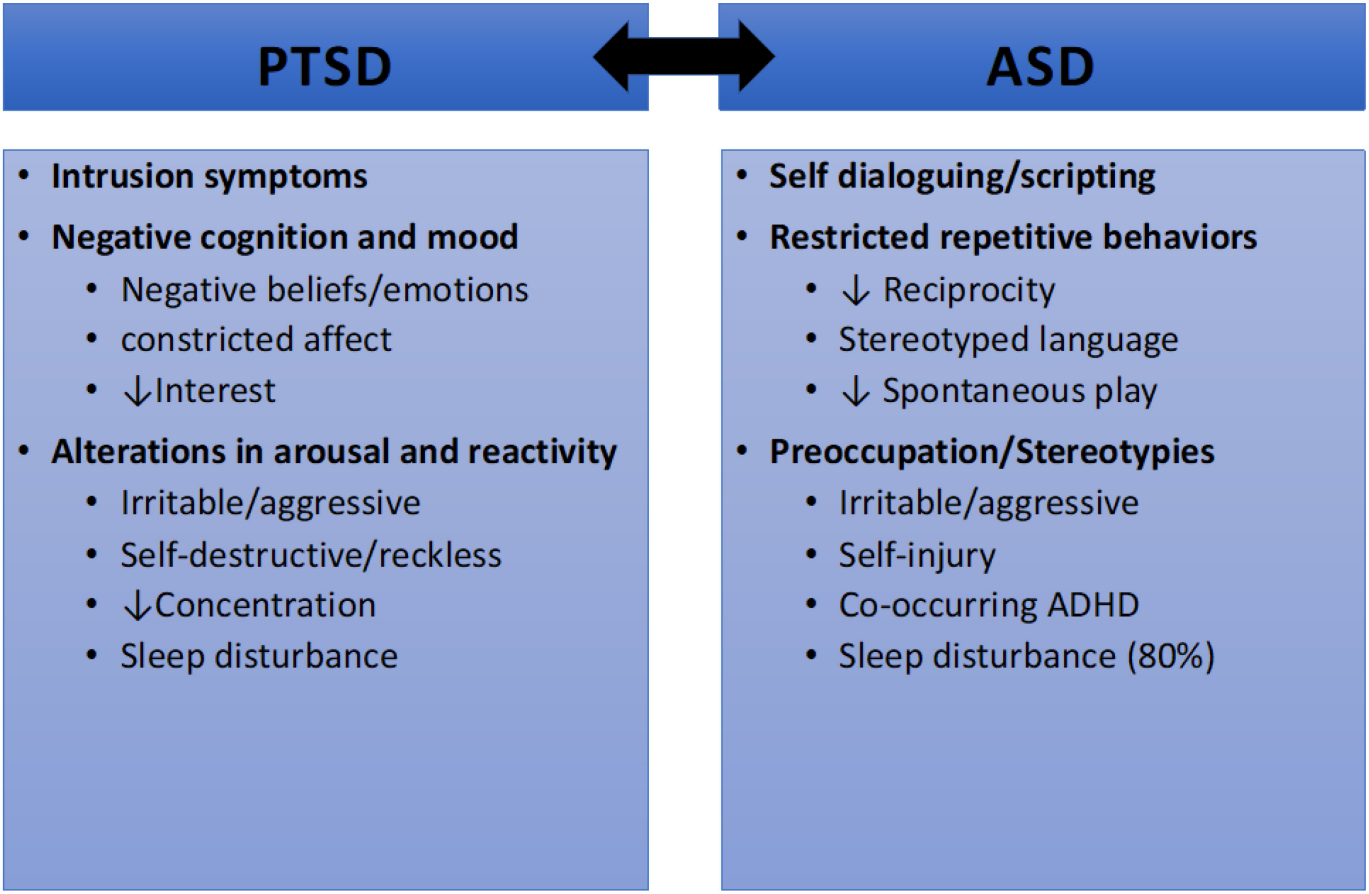
Symptomatic overlap between PTSD and ASD (8).

Not only do the deficits in social communication experienced by individuals with ASD increase the difficulty of reporting symptoms of PTSD, some symptoms of ASD can also appear similar to symptoms of PTSD (**Figure 1**) (9). While individuals with PTSD can experience intrusions such as flashbacks and dissociation, individuals with ASD can experience self-dialoguing and scripting which can present similarly to intrusions (31). Individuals with PTSD will experience negative mood and cognition. Meanwhile, individuals with ASD will demonstrate reduced reciprocity, stereotyped language and reduced spontaneous play (8, 13). Individuals with PTSD may also exhibit alterations in arousal and reactivity. Individuals with ASD can present as irritable or with aggressive mood, self-injury, and sleep disturbance independent from the presence or absence of PTSD (31).

In addition, co-occurring ASD and PTSD may present with overlapping symptoms (9). For example, individuals with ASD and PTSD may experience recurrent and intrusive recollections which manifest in repetitive play (8). They may demonstrate avoidance or emotional numbing with efforts to avoid trauma reminders, decreased interest in participating in previously enjoyed activities, and restricted affect (8). Hyperarousal may present simply with sleep disturbance, angry outbursts, difficulty concentrating, hypervigilance, or increased startle reaction (8). It has also been observed that young children with ASD and PTSD may manifest: new aggression, oppositional behavior, regression in developmental skills (toileting, speech), new separation anxiety, new fear not obviously related to traumatic event (dark, going to bathroom alone).Diagnostic challenges to identifying PTSD in children with ASD are further complicated by the high rate of psychiatric co-occurrence in individuals with ASD (4). Mutluer et. al., reviewed research on the prevalence of psychiatric co-occurrence in children and adolescents with ASD and their results are summarized in **Table 1**. Most reviewed studies focused on diagnoses in early childhood and latency age with a significant gap identified in prevalence studies focused on adolescents. Most significantly, ADHD was found to have the highest rates of co-occurrence in individuals with ASD at 26.2% (4), which can further complicate the presentation of alterations in arousal and reactivity in these individuals.

**Table 1:** Psychiatric co-morbidity with ASD prevalence and clinical presentation (4).

Psychiatric Co-Morbidity	Prevalence of Co-Morbidity in Children with ASD	Clinical Presentation of Co-occurrence with ASD
Intellectual Disability	22.9%	Defined in terms of measurement tools, WISC-4 and FSIQ, however did not incorporate overall adaptive functioning and IQ scores alone cannot point to the severity level of ID. Authors noted that there is “poor testability” of subjects with comorbid ASD, which led to significant variance in results. Adaptive functioning, meanwhile, was not reported.
Attention-Deficit Hyperactivity Disorder	26.2%	Inattention, hyperactivity-impulsivity, impairments in activities of daily living, social adaptation, behavior problems
Internalizing Disorders Anxiety Disorder Depression	11.1% 2.7%	Social communication problems, sensory aversions, disruptive emotional dysregulation, inflexible adherence to routines, difficulty tolerating change.
Sleep disorder	19.7%	Heightened daytime cognitive, adaptive, and behavioral problems.
Disruptive Disorder	7%	Oppositional defiant disorder, conduct disorder, and disruptive behavior problems.
Bipolar Disorder	2%	Prevalence increased as older age groups were included in the analysis.
Obsessive-Compulsive Disorder	1.8%	Restrictive repetitive behaviors associated with ASD tend to be ego-syntonic compared to ego-dystonic nature of OCD symptoms.
Psychosis	0.6% (1.1% among adolescents)	Behavioral phenotypes of known genetic conditions such as 22q11 deletion syndrome possibly connected to greater likelihood for the identification of psychosis.

Given the high level of diagnostic complexity in individuals with ASD and co-occurring psychiatric disorders (4), it is recommended that clinicians caring for these children work in collaboration with parents and a range of providers including primary care clinicians, speech pathologist, occupational therapists, and teachers or school counselors. Obtaining collateral information from those providers allows clinicians to incorporate multiple perspectives of the child’s functioning and offers opportunities to provide psychoeducation on responses to trauma. In addition, trauma-focused cognitive behavioral therapy (TF-CBT) is one evidence-based trauma-specific intervention that can be adapted to treat individuals with ASD (14). Eye Movement Desensitization and Reprocessing (EMDR) therapy is another preferred method for treating PTSD in the general population. One study examining the use of EMDR in adults with ASD and a history of trauma did show significant improvement in PTSD symptoms when compared to treatment as usual (Impact of Event Scale-Revised: *d*=1.16), however study limitations included small sample size (n=27), lack of control group, inability to blind participants, and researcher bias, as the therapists providing the intervention also completed the measures (32). The use of EMDR in children and adolescents with PTSD has been shown to reduce scores on the PTSD symptom scale from 60 ± 8.7 to 24 ± 10.1, *p*= 0.001) in this age group, though the study was limited by small sample size (n=30), absence of a control group and lack of follow-up measurements beyond six weeks (33). Despite the limitations of these studies, it is possible the EMDR would be beneficial in children and adolescents with co-occurring ASD and PTSD, though further research would be necessary (32, 33).

## Disaster response and intervention

The COVID-19 pandemic impacted community resources, access to programs, and interrupted supports for families, presenting a natural disaster that threatened the physical safety of the global population. Vulnerable populations such as people with disabilities, developmental, behavioral, and mental health disorders were at higher risk for poor physical and mental health outcomes that resulted from the COVID-19 pandemic and public health measures to address it (www.cdc.gov/disasters/covid-19, 9/1/2023) (27).

The impact of disasters on children with autism is not well-studied. Valenti et al. published a 2011 study that examined the adaptive behavior of participants with ASD one year after their exposure to the 2009 earthquake in L’Aquila, Italy compared with an unexposed peer group with ASD. The researchers showed that adaptive behavior in the exposed individuals declined during the first months after the earthquake (*p*<0.01). The COVID-19 pandemic was unique from other natural disasters because of the wide exposure to its impact. An ongoing Yehuda Science Foundation COVID-19 study suggested that mental health consequences are primarily found among adults with the most direct exposures to the impact of the COVID-19 pandemic. There are no current parallel studies focused on mental health outcomes for children (34).

Studies of children exposed to prolonged war note that no single exposure alone can determine whether a child will later develop PTSD. However, exposures that lead to multiple other exposures (i.e.: siege exposure) can place the child at risk for all trauma-related outcomes (35). As shown in the ecological analysis above, children and adolescents with ASD and/or ID are at an increased risk of trauma exposure, financial strain, loss of community supports, all of which make them more vulnerable to trauma that can lead to PTSD. There are ongoing efforts to study the global predictors of mental health outcomes for children during the pandemic, however an analysis of these risk factors is not currently available (36). Even so, it is clear that this vulnerable population of children requires special attention so that they receive accurate mental health diagnosis and appropriate treatment (35).

In the case of a natural disaster, immediate intensive post-disaster intervention has been shown to allow children and adolescents with ASD to trend toward recovery of adaptive functioning (37). With this in mind, children with ASD and ID would benefit from collaborative care where changes in behavior are discussed and shared with providers. Evidence-based treatment for children with ASD and PTSD include trauma-focused cognitive behavior therapy that is adjusted to the developmental and skill level of the patient (14). This intervention is best received with collaboration among all providers for the patient as well as family, school, and primary care physicians.

## Conclusion

While children with ASD and ID have long been understood to experience higher rates of trauma and maltreatment, the detection of PTSD among other sequelae of trauma exposure has remained diagnostically challenging in this population. The recent COVID-19 pandemic presented a challenge to all vulnerable populations and provides the opportunity to better understand stress response as well as the importance of community supports for families of children with ASD. Given the significant stressor heralded by the COVID-19 pandemic, loss of supports, family stress, and increased risk of domestic violence during this period, special attention to mental health sequelae will be essential in identifying PTSD in the clinical setting and providing appropriate treatment. Further investigation to better understand risk factors, prevalence, and treatment of trauma reactive disorders in this population will be essential.

## Author contributions

SP: Writing – original draft, Writing – review & editing. YD: Writing – original draft, Writing – review & editing.
